# Development of the Home Environmental Scale of Accessibility Instrument for Spain

**DOI:** 10.3390/clinpract14030089

**Published:** 2024-06-12

**Authors:** Estíbaliz Jiménez-Arberas, Gemma Ruíz Varela, Feliciano Francisco Ordoñez Fernández, María Isabel Fernández Méndez

**Affiliations:** 1 Faculty Padre Ossó, Occupational Therapy Degree, 33001 Oviedo, Spain; estibaliz@facultadpadreosso.es (E.J.-A.); isabelf@facultadpadreosso.es (M.I.F.M.); 2 Faculty Educational Sciences, University Alfonso X el Sabio, 28691 Madrid, Spain; gemmaruiz@uax.es

**Keywords:** home accessibility, disability, universal accessibility, analysis factorial, environment, mobility, assistive technology

## Abstract

Background: Universal accessibility is one of the most active lines of intervention for people with disabilities and older adults. This accessibility has become a topic of growing interest regarding home access and use. Therefore, the main objective of this study was to create and validate a home assessment tool: the HESA II. Methods: The study was conducted in four phases: (1) agreement on variables by an expert panel; (2) development of 90 items according to the AOTA framework; (3) pilot test with n = 20; and (4) final study with 156 subjects where confirmatory factor analysis was performed. Results: The tool consisted of 85 items divided into five subscales related to each of the main spaces of Spanish homes: living room; kitchen; bedroom; and bathroom. Conclusions: The tool demonstrates good psychometric properties of reliability. The HESA II assesses home accessibility based on limitations in activity and participation restriction of the evaluated person as per the International Classification of Functioning, Disability, and Health rather than on a diagnosis, making it applicable to a wide range of groups.

## 1. Introduction

Various organizations and entities have made significant efforts to urge different states and governments to consider accessibility and universal design, which is currently a strategic focus in Spain [1]. In Spain, universal accessibility has become one of the most active and forefront areas at the governmental and legislative levels. Universal accessibility is defined as the condition that environments, products, and services must meet to be understandable, usable, and practicable by all individuals [2]. The home is the environment where we carry out the majority of our activities of daily living (ADLs), which are understood as “the everyday activities that people do as individuals, in families, and with communities to occupy time and bring meaning and purpose to life. Occupations include things people need to, want to, and are expected to do” [3] p. 2.

Home modifications were mainly focused on architectural changes, especially in the bathroom, master bedroom, and kitchen areas, where the majority of activities of daily living (ADLs) take place. In a systematic review conducted by Cho et al. (2016) [4], the conclusion drawn was that interventions aimed at enhancing the accessibility of homes can indeed have positive effects. However, it was noted that standardized tools with a solid foundation are needed to effectively measure these effects. Additionally, it appears that positive outcomes are also reported by caregivers and family members. There is already scientific literature that has investigated the relationship between self-perceived limitations in home accessibility and limitations in the occupational performance of ADLs and instrumental activities of daily living (IADLs) [5,6], as well as in the reduction of falls among older adults [7,8].

Universal accessibility refers to the design of products, environments, programs, and services that can be used by all people to the greatest extent possible without the need for special adaptations or designs. This definition is supported by several official bodies and international documents. For example, the United Nations Convention on the Rights of Persons with Disabilities (CRPD) [9] states that accessibility is a fundamental principle for the full participation in society of persons with disabilities. Likewise, the World Health Organization (WHO) in its International Classification of Functioning, Disability and Health (ICF) [10] (https://www.who.int/standards/classifications/international-classification-of-functioning-disability-and-health (accessed on 10 May 2024) defines accessibility as the ability to access and use products, services, and environments by all people, in conditions of equality and security. Universal accessibility is one of the most common intervention strategies in occupational therapy, including environmental modification, which involves assessment, selection, provision, education, and training in the use of high- and low-tech assistive technology; the application of universal design principles; and recommendations for changes to the environment or activity to support the client’s ability to engage in occupation [11] p. 60. Although there is legislation regulating the physical accessibility of public buildings, websites, etc., it was not until 2019 [12] that the digital accessibility law was approved as a standardization of European accessibility. Each country, and even autonomous communities within countries, has legislation regarding home accessibility, but Spain has deeply ingrained physical characteristics rooted in its culture compared to other countries. There are several assessment tools for home accessibility, including the Housing Enabler Instrument [13]; Home and Community Environment Instrument (HACE) [14]; Home Assessment of Person-Environment Interaction (HoPE) [15]; Comprehensive Assessment and Solution Process for Aging Residents (CASPAR) [16]; and the Home Environmental Assessment Protocol (HEAP) [5].

In Spain, according to the National Institute of Statistics, more than 1,300,000 elderly people live alone, 77% of whom are women [17]. In this survey, it was verified that in the year 2023, 79.64% of homes were inaccessible in our country. Although each autonomous community has different regulations for the parameters of accessibility in terms of measures and solutions, sometimes, this current legislation is merely indicative. And as social and health professionals, we must take into consideration the individual characteristics and cultural specificities of our country.

The European Disability Strategy 2010–2020 indicates that the convenience of regulating accessibility in different areas has a great impact on the autonomy of people with disabilities to access equal conditions with other service users. By March 2021, the European Commission adopted the strategy on the rights of people with disabilities 2021–2030. Transferring these general principles to state legislation on accessibility, it was initially established through Law 13/1982, of April 7, on the social integration of people with disabilities, which laid the foundations for the elimination of barriers, architecture, and communication and the promotion of accessibility [18]. It was developed mainly through Law 51/2003, of December 2, on equal opportunities, non-discrimination, and universal accessibility of people with disabilities, issued under the exclusive jurisdiction reserved to the State to guarantee the equality of all Spaniards in the exercise of rights and in the fulfillment of constitutional duties. The regulations that regulate accessibility in homes are Law 8/2013, of 26 June, on urban rehabilitation, regeneration, and renewal [19]. This law establishes measures to promote accessibility in buildings and homes, especially for people with disabilities or reduced mobility. Although the Urban Rehabilitation Law addresses aspects of accessibility in homes, it may lack specificity regarding the adaptation of the environment to facilitate the performance of specific activities of daily living. To give an example, despite taking into consideration basic parameters such as circulation (passage space, rotation space, and turning space), it does not consider transfers at home as if they are carried out laterally or if you need a support product or a person to help you. It also does not consider the scope; that is, it does not regulate the approach space, and this facilitates simple actions such as touching, grabbing, and even more complex activities such as cooking.

Despite the mentioned instruments, none of them have been translated and adapted to the Spanish population. Additionally, except for the Housing Enabler [13], they do not evaluate accessibility issues independently of the person’s limitations and capacities or their diagnosis, focusing the problem on the individual rather than the environment as described by the International Classification of Functioning, Disability, and Health [10]. This study builds upon the vertical accessibility assessment tool Home Environmental Scale of Accesibility_HESA I for the home, and the present study introduces the HESA II for horizontal accessibility within it. The overall aim was to create an instrument (Home Environmental Scale of Accessibility II) to measure the limitations in the activity of each person related to the horizontal accessibility of their home.

## 2. Materials and Methods

### 2.1. Design

The design employed was a construct validation through confirmatory factor analysis. This methodological design allows for the assessment of the reliability and validity of each item rather than global values, as in other models. This analysis enables the optimization of the scale construction and adaptation process.

### 2.2. Procedure

For the development of the HESA II scale, the following steps were undertaken:

1. Eight meetings were conducted with the expert team (n = 8), during which the determination of variables to be studied regarding home accessibility was agreed upon.
2. A total of 90 items were developed, covering a wide range of tasks within the home. The scale’s structure, for better management and interpretation, is divided into several subscales corresponding to different spaces/areas of analysis within the home. The theoretical framework of the AOTA [11] was used, along with the DALCO requirements (ambulation, apprehension, localization, and communication) [12]. The DALCO criteria define the conditions of accessibility in terms of the different activities that people commonly carry out, moving, communicating, reaching, understanding, using, and manipulating.
3. The items were focused on five spaces: living room, kitchen, bedroom, bathroom, and other areas of the home referred to as Specific Elements of Indifferent Space Usage. These spaces were selected due to their cultural relevance as typical areas in Spanish households.
4. Pilot Test: A pilot test was conducted with a total of 20 participants. A non-probabilistic ex post facto sampling method was employed to select the entire sample, including that which was utilized in the pilot study, with the final sample for analysis consisting of 156 individuals from the Principality of Asturias, excluding the 20 participants from the pilot study [20,21,22].
5. For the selection of the samples used, the following process has been followed. For the sample calculation of the pilot study, it is recommended to include between 30 and 50 cases. In our pilot study, we included 20 cases due to the difficulty in obtaining a homogeneous population by applying strategies to minimize the number of participants. To achieve this, we conducted a random selection to reduce the variability of the measurements [23]. In articles that analyze the number of cases as one of the standards related to factorial analysis, it is argued that the sample size should not be less than 50, and it is stated that it is preferable for this size to be greater than 100 [22]. Arrindell and van der Ende (1985) concluded that a stable factorial solution is possible when the sample size approaches 20 times the number of factors [21]. These studies support the use of a sample size smaller than 200, which is indeed considered an ideal number [20]. In our case, due to the difficulty of accessing a sample tailored to the needs of our study, we aim to achieve at least 20 times the number of factors, with a maximum of 5, surpassing 100, and therefore, we have a sample within the necessary margins. Age was analyzed in intervals, with 54% of participants being over the age of 65, predominantly women (63.3%), with a socio-economic level between EUR 1000 and 2000 (42.25%), residing mostly in urban central areas (52.82%; 16.2%) and living in apartment blocks (67.6%). See Table 1 for the descriptive data of the sample used in the study.
6. The study was approved by the Bioethics Committee of the Principality of Asturias (number 2020.091). In the questionnaire provided to the participants, the first question was related to consenting to the publication of the data anonymously, following the current Organic Law 3/2018, of December 5, on the Protection of Personal Data and Guarantee of Digital Rights.

### 2.3. Instrument

The initial questionnaire for analysis was a Likert-type scale (1932) [24] consisting of 90 items. The first of these was the construction of the preliminary items for which 8 meetings were held with the team of experts (n = 8), where it was agreed to determine the variables that they wanted to study in relation to the accessibility of the home (n = 5 occupational therapists; n = 1 psychologist, n = 1 quantity surveyor, and n = 1 social worker). The methodology used to determine this measurement instrument in its validation phase was an Attribute Agreement Analysis, which determines the degree of agreement among judges [25]. The Attribute Agreement allowed for the evaluation of the uniformity of responses made by the group of evaluators. The analysis used was Fleiss’ Kappa, with acceptance values set at 0.75 and those below 0.40 eliminated [26]. Once the analysis was completed, the process continued with a pilot study involving 20 individuals. After analyzing the difficulties encountered by the experts and the pilot study, the 90 items that were definitively passed on to the study participants were drafted, following the analysis of agreement. With this process, a consistent selection of items for the final questionnaire was maintained [27,28].

### 2.4. Data Analysis

A consistency analysis of the questionnaire used in its final configuration was conducted using Cronbach’s α and McDonald’s ω (ordinal reliability), both for the total scale and for sub-scales.

The validation process was carried out in two phases: an Exploratory Factor Analysis and a Confirmatory Factor Analysis using the JASP software 17, a free program that allows for assessments with various fit estimators [29]. The advantage of using JASP for analysis is that it allows for the interpretation of the proportion of variance for each of the factors, as well as the use of polychoric correlations, which are most suitable for Likert-type questionnaires [30,31].

The chosen estimation method for the fit function was the Diagonal Weighted Least Squares (DWLS) estimator. This choice aims to obtain a saturation vector that reproduces the observed matrix as closely as possible, minimizing potential errors. The DWLS estimator is recommended when the assumption of normality is not maintained, and there are not large samples [32]. For these models, this estimator was proven to be more robust than maximum likelihood (ML) [33,34]. Confirmatory Factor Analysis was evaluated using global fit indices. Within the global model, we followed the absolute fit model and incorporated two measures of incremental fit: NFI and CFI, which reinforce the model’s fit [35]. The indices selected for this analysis are determined as follows:

- Chi-square: It is estimated that this value should be greater than 0.05. This indicator is highly sensitive as it follows a chi-square distribution [36,37]. Therefore, it is recommended to complement the results with other goodness-of-fit indices, among which the most commonly used one is the Root-Mean-Square Error of Approximation (RMSEA) [37,38,39]. This measure helps determine the significance of the model.
- Root-Mean-Square Error of Approximation (RMSEA): For this index, scales with values below 0.05 are considered valid [40,41,42]. Adequate values establish symmetry in the scale.
- Goodness-of-Fit Index (GFI): This index indicates the variability explained by the model. Values above 0.90 are considered good fits [43].
- Normed Fit Index (NFI): Values close to one are recommended [44].
- Comparative Fit Index (CFI): This index indicates a good scale fit for values close to one, preferably above 0.95 [43,44].

## 3. Results

The group of experts used the DALCO [45] criteria as a reference, since the current legislation in force regarding home accessibility does not take into consideration the motor and processing skills necessary for actions that take place at home.

For example, according to the AOTA framework, this includes the observation of motor skills, understanding these as “the group of performance skills that represent small, observable actions related to moving oneself or moving and interacting with tangible task objects (e.g., tools, utensils, clothing, food or other supplies, digital devices, plant life) in the context of performing a personally and ecologically relevant daily life task” [46] p. 331. Positioning the body (stabilizing, aligning, and positioning) and obtaining (reaching, bending, gripping, and manipulating) are included in the DALCO ambulation criteria. Holding objects, moving self and objects (coordinating and moving) are skills included in the application of the DALCO criteria. Along these same lines, localization and communication are included in the processing skills of the AOTA framework itself, understood as “Process Skills—“Process skills are the group of performance skills that represent small, observable actions related to selecting, interacting with, and using tangible task objects (e.g., tools, utensils, clothing, food or other supplies, digital devices, plant life); carrying out individual actions and steps; and preventing problems of occupational performance from occurring or reoccurring in the context of performing a personally and ecologically relevant daily life task” [46] (pp. 336–337).” These processing skills can be sustaining performance (attending and heeling...); applying knowledge (choosing and using...); or adapting performance (adjusting and noticing) included in the localization and communication of the DALCO criteria.

The distribution of the rooms was agreed upon by all the experts due to the combination of several environmental and personal factors such as cultural and social tradition. In Spain, the kitchen and living room are the central spaces of the house where family and direct personal relationships meet, socialize, and share meals, and the bathroom and bedrooms are essential for some essential daily living activities such as self-care, hygiene, shower or rest and sleep, among others, since they require individual privacy. Although the Technical Building Code in Spain does not specify the distribution of spaces in homes, it does establish requirements related to habitability and other areas that influence the design and distribution of homes (for example, the CTE establishes requirements for the installations of plumbing, sanitation, electricity, heating, which includes distribution and habitations [47].

The total scale is structured into five spaces. Subscales include the following:

1. Space corresponding to the living room;
2. Space corresponding to the kitchen;
3. Space corresponding to the bedroom;
4. Space corresponding to the bathroom;
5. Specific elements of use regardless of space.

**Results obtained from the different spaces of analysis**

1. Space corresponding to the living room.

**Figure 1:**
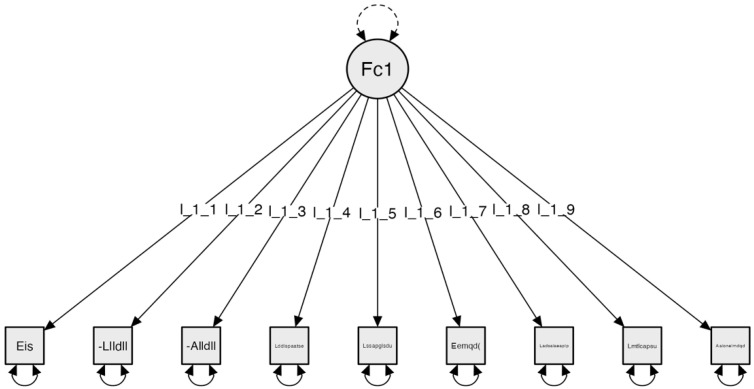
Subscale 1 Model. Living Room Space (ESES). The factor is configured as follows: Factor 1: Living Room Accessibility: (Eis): There is sufficient lighting (I_1_1); (Llldll): Able to locate light switches; (Alldll): Able to reach light switches; (Lddlspaatse): The layout of the living room allows access to all its space; (Lssapglsdu): Floors are suitable to ensure user safety; (Eemqdem(cs)): Presence of movable elements that hinder movement (cables, chairs, etc.); (Ladsalaeaaalp): The height from the floor to the seats is suitable for the individual; (Lmtlcapsu): The table meets the necessary conditions for its use; (Alonelmdqd): Able to reach necessary items in the furniture available.

These are the results of the subscale corresponding to the living room. The initial configuration consisted of 10 items. Following this initial Exploratory Factor Analysis (EFA) using the JASP program (2019) [3], a result of one factor formed by nine items, which exceeded 0.4 variance, was obtained. With this result, Confirmatory Factor Analysis (CFA) was conducted, and the obtained results for the indicators are as follows:

Confirmatory Factor Analysis (CFA) using JASP v. 0.11.1, 2019. Results of the fit indices: Absolute model fit indices and incremental model fit indices: Chi-square χ²(45) = 138.739, *p* = 0.968; RMSEA ≤ 0.001; GFI = 0.997; NFI = 0.886; and CFI = 0.999 (Figure 1).

*Internal consistency (reliability):*

Reliability calculations for the definitive subscale yielded Cronbach’s α of 0.741 and McDonald’s ω of 0.784.

2. Space corresponding to the kitchen

Results of the subscale corresponding to the kitchen revealed a total of two factors. Starting with an initial configuration of 22 items, this initial analysis yielded two components, with all 22 items surpassing 0.4 variance. With this result, Confirmatory Factor Analysis (CFA) was conducted. *The following item does not provide relevant information: “Existen alfombras que lo dificulten” (“There are carpets that hinder movement”). This item appears to be redundant and can be assessed through the item that measures movable elements.

**Figure 2:**
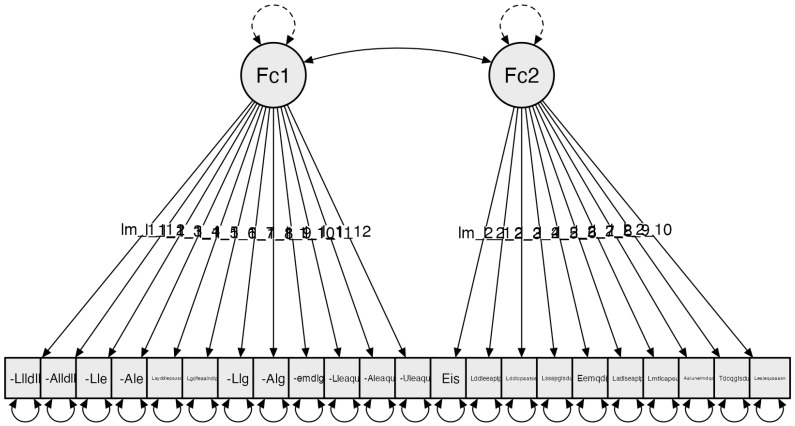
Subscale 2 Model: Kitchen (C). The factors are configured as follows: Factor 1: Location and Reach: (Llldl): Locates light switches (Im_1_1); (Alldll): Reaches light switches; (Lle): Locates electrical outlets; (Ale): Reaches electrical outlets; (Lgdfeaalndlp): The sink faucet is suitable for the person’s needs; (Llg): Locates the sink faucet; (Alg): Reaches the sink faucet; (Uemdg): Uses the sink faucet; (Layddlepsusd): The height and dimensions of the countertop allow for easy use; (Llaqu): Locates auxiliary elements used; (Aeaqu): Reaches auxiliary elements used; (Ueaqu): Uses auxiliary elements used. Factor 2: Distribution, Elements, and Spaces: (Eis): There is sufficient lighting (Im_2_1); (Lddeeaalp): The distribution of electrical outlets is suitable for the person; (Lddlcpaatee): The kitchen layout allows access to all the space; (Lssapglsdu): Floors are suitable to ensure user safety; (Eamqd): There are movable elements that hinder (cables, chairs, etc.); (Ladlseaplp): The height of the chairs is suitable for the person; (Lmtlcapsu): The table meets the necessary conditions for its use; (Aalunelmdqd): Reaches necessary utensils in the available furniture; (Tdcqglsdu): Type of kitchen that ensures user safety; (Lea€qusaasn): The auxiliary elements (appliances, etc.) used are suitable for their needs.

Confirmatory Factor Analysis (CFA) results, adjusting the model using absolute model fit indices and incremental model fit indices: Chi-square χ²(231) = 1182.902, *p* = 0.999; RMSEA ≤ 0.001; GFI = 0.957; NFI = 0.936; and CFI = 0.999 (Figure 2).

*Internal consistency (reliability):*

Reliability calculations for Subscale 2, Kitchen Mobility, yield Cronbach’s α and McDonald’s ω of 0.921 and 0.926, respectively.

3. Space corresponding to the bedroom

**Figure 3:**
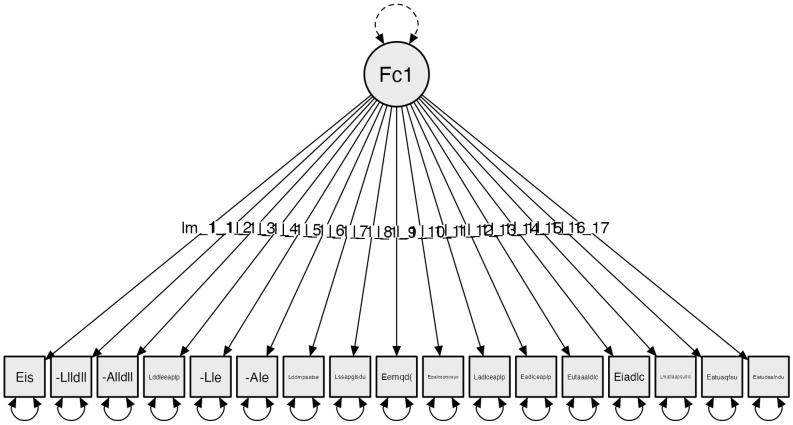
Subscale 3 Model: Bedroom (D). The factor is configured as follows: Factor 1: Bedroom Mobility: (Eis): There is sufficient lighting (Im_1_1); (Llldll): Locates light switches; (Alldll): Reaches light switches; (Lddleeaplp): The distribution of electrical outlets is suitable for the person; (Lle): Locates electrical outlets; (Ale): Reaches electrical outlets; (Lddmpaa): The furniture layout allows access to all its space; (Lssapglsdu): Floors are suitable to ensure user safety; (Eemqd(cs)): There are movable elements that hinder (cables, chairs, etc.); (Eptalcsphcsyc): Transfer to/from the bed can be done safely and comfortably; (Ladlceaplp): The height of the bed is suitable for the person; (Eadlceaplp): The width of the bed is suitable for the person; (Eutaaaldlc): The user has access to both sides of the bed; (Eiadlc): There are switches accessible from the bed; (Lmatlaapsudlc): Auxiliary tables have the appropriate height for use from the bed; (Elatuaqfsu): The wardrobe(s) have(s) an opening that facilitates use; (Eatudaalndu): The wardrobe has a layout suitable for the user’s needs.

The Exploratory Factor Analysis of the third subscale, Bedroom Mobility, establishes a one-factor model. Starting with an initial configuration of 17 items, this initial analysis results in a one-factor model with 17 items exceeding 0.4 variance. With this result, Confirmatory Factor Analysis (CFA) was conducted. The obtained results for the indicators are as follows: Confirmatory Factor Analysis (CFA), adjusting the model using absolute model fit indices and incremental model fit indices: Chi-square χ²(119) = 76.395, *p* = 0.999; RMSEA ≤ 0.001; GFI = 0.921; NFI = 0.810; and CFI = 0.999. (Figure 3).

*Internal consistency (reliability):*

Reliability calculations for Subscale 3, Bedroom Mobility, yield Cronbach’s α and McDonald’s ω of 0.831 and 0.856, respectively.

4. Space corresponding to the bathroom

**Figure 4:**
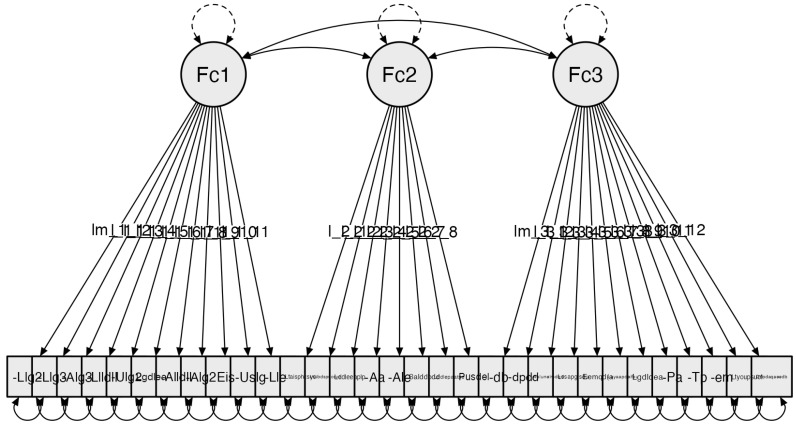
Subscale 4 Model: Bathroom Mobility (B). The factors are configured as follows: Factor 1: Location, visibility, and reach: (Llgdb): Locates the shower/bathtub faucet (Im_1_1); (Algdb): Reaches the shower/bathtub faucet; (Ulagdb): Uses the shower/bathtub faucet; (Lgdlea): The sink faucet is suitable; (Llgl): Locates the sink faucet; (Algl): Reaches the sink faucet; (Ulgl): Uses the sink faucet; (Eis): There is sufficient lighting; (Llldll): Locates light switches; (Alldll): Reaches light switches; (Lle): Locates electrical outlets. Factor 2: Movement in space and distribution of elements: (Ltaisphcsyc): Transfer to the toilet can be done safely and comfortably (I_2_1); (Ltalbdsphcsyc): Transfer to the bathtub/shower can be done safely and comfortably; (Lddleeaalp): The distribution of electrical outlets is suitable for the person; (Ale): Reaches electrical outlets; (Pusdel): Can use the sink without difficulty; (Aal): Suitable height (sink); (Salddpddb): Dimensions of the shower/bathtub are adequate; (Lddlepaatse): The distribution of elements allows access to all its space. Factor 3: Features and availability of bathroom elements: (Ddb): Has a bathtub (Im_3_1); (Ddpdd): Has a shower tray; (Ddpdaqaeedb): Has assistive products that adapt to the bathroom environment; (Alunelmdqd): Reaches necessary utensils in the available furniture; (Lssapglsdu): Floors are suitable to ensure user safety; (eemqdemta): There are movable elements that hinder movement (towel racks/mats); (Ltyoupsucf): Towel racks and other utensils allow easy use; (Layaapdeifsu): The height and access to toilet paper from the toilet facilitate its use; (Lgdldbea): The shower/bathtub faucet is suitable; (Eltlpa): The sink has the appropriate depth; (Eltp): The sink has a pedestal; (Eleeeem): The sink is recessed into the furniture.

The Exploratory Factor Analysis of the fourth subscale, Bathroom Mobility, begins with an initial configuration of 34 items. Following this exploratory analysis, a result of three components with all items exceeding 0.4 variance is obtained. With this result, Confirmatory Factor Analysis (CFA) is conducted. Due to the specificity of the elements in this subscale, the Unweighted Least Squares (ULS) estimator is used instead of the DWLS. When working with items/variables that do not follow a specific distribution, the ULS estimator can be used in policoric correlation matrices, allowing for model adjustment [35,39,48,49,50]. The obtained results for the indicators are as follows: Confirmatory Factor Analysis (CFA), adjusting the model using absolute model fit indices and incremental model fit indices: Chi-square χ²(431) *p* = 0.931 = 1062.015; RMSEA ≤ 0.001; GFI = 0.907; NFI = 0.671; and CFI = 0.999 (Figure 4). Following this analysis, the factor adjustment results in a total of 31 items.

*Internal consistency (reliability):*

Reliability calculations for Subscale 4, Bathroom mobility (final version), yield Cronbach’s α and McDonald’s ω of 0.841 and 0.856, respectively.

5. Specific elements of use regardless of space

**Figure 5:**
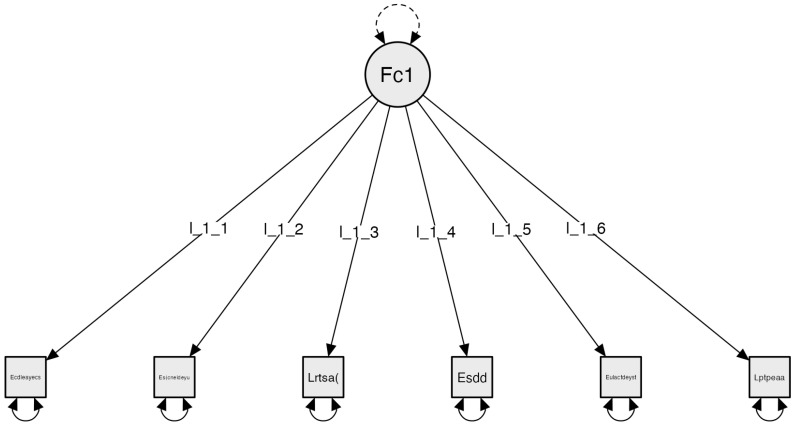
Subscale 5 Model: Specific Elements of Space-Indifferent Use (E3). The factor is configured as follows: Factor 1: Functional and Signage Elements: (Ecdleaecs): The light switch panel is accessible and properly labeled; (Esecneldeys): There is signage (if necessary) in different areas of use; (Lrtsa): Radiators have appropriate signage (open/closed); (Esdd): Home automation systems are available; (Eulactdeyst): There is an accessible list with emergency phone numbers and technical services; (Lptpeaa): Toxic/hazardous products are properly stored.

The Exploratory Factor Analysis of the fifth subscale, Specific Elements of Space-Indifferent Use, establishes a one-factor model. Starting with an initial configuration of 6 items, this initial analysis results in a one-factor model with all 6 items exceeding 0.4 variance. With this result, Confirmatory Factor Analysis (CFA) is conducted. The obtained results for the indicators are as follows: Confirmatory Factor Analysis (CFA), adjusting the model using absolute model fit indices and incremental model fit indices: Chi-square χ²(9) = 11.567, *p* = 0.239; RMSEA = 0.049; GFI = 0.991; NFI = 0.840; and CFI = 0.955 (Figure 5).

*Internal consistency (reliability):*

The reliability calculations for Subscale 5, Specific Elements of Space-Indifferent Use, yield Cronbach’s α and McDonald’s ω of 0.615 and 0.641, respectively.

The final scale consists of five subscales with a total of 85 items. Reliability calculations for the entire scale yield a result of α = 0.946 and ω = 0.938. These results, combined with the psychometric validation conducted, allow for establishing adequate reliability and validity for the use of the scale.

## 4. Discussion

Occupational therapists who visit homes to assess their accessibility often use different tools to evaluate the level of occupational performance of end users [51]. Despite several assessment tools for home accessibility existing, they are not validated for the Spanish context, and most of them assess accessibility based on diagnosis and difficulties rather than individual accessibility.

The current law on home accessibility may not exhaustively address people’s individual needs in terms of adapting the home environment for activities such as personal hygiene, food preparation, and mobility in confined spaces, among others. Proof of this is that according to data from the Statistics National Institute, only older people who live alone in our country find that 80% of their housing is inaccessible; along these same lines if we base ourselves on the FOESSA report, “In 2018, housing problems were the essential feature of social exclusion processes” [52] p. 14.

The law could benefit from greater integration of professionals such as occupational therapists in the planning and implementation of accessibility measures, thus ensuring a more personalized and effective adaptation of the environment to the needs of each individual.

The main objective of this study was to validate an instrument created from expert analysis capable of measuring the activity limitations of each individual regarding home accessibility. The results of the psychometric and confirmatory factor analyses conducted showed an adequate factorial structure, reliability, and validity of the instrument.

On one hand, the reliability calculations were acceptable in all cases. Thus, Cronbach’s alpha (α) is shown to be above the minimum recommended value of 0.70 [53], with other authors [54] suggesting that in confirmatory studies, it should fall between 0.7 and 0.8. Regarding omega (ordinal alpha, ω McDonald), a reliability value between 0.70 and 0.90 is considered acceptable [55]. The results of the Confirmatory Factor Analysis with the final instrument reduced to 85 items were excellent in all indicators. Hence, it can be affirmed that HESA II demonstrates robust construct validity.

For Confirmatory Factor Analysis, standard steps involve starting with the initial structure resulting from Exploratory Factor Analysis, then refining hypotheses and correcting deficiencies found in the exploratory analyses [56]. Initially, exploratory analysis is conducted to examine the dataset without any preconceived hypotheses about its structure, with the results guiding the model [57]. This initial analysis allowed for the establishment of an initial structural hypothesis. Considering the Likert-type scoring of the instrument, JASP (2019) [3] facilitated the use of polychoric correlation matrices, already establishing a definitive number of factors and items.

The adjusted model was conducted using the open-source software JASP (2019) [3]. The advantage of this program was its ability to use different factor estimation models, not just the standard ML of SPSS but also ULS and DWLS. The estimation model that best fit the analysis was DWLS, as it allowed for maintaining all possibilities of analysis from the initial theoretical postulates of the instrument’s construction. Additionally, ULS was added to one of the models as it better suited the type of items used. The goodness-of-fit indices used included global fit indices such as chi-square, RMSEA, and GFI. Furthermore, CFI and NFI were added, which are incremental or comparative fit indices that help determine the definitive model compared to the independent or non-relationship model between variables [34,58].

Regarding other assessment tools developed for the evaluation of home accessibility, it should be noted that the analyses used in the HoPE [15], HACE [14], and Housing Enable Screening Tool [13] base the validity of the structure of their scales on content validation [59]. However, construct validity is considered the most acceptable system with the most evidence for the validation process [60,61], Regarding the number of cases used in the validation and reliability process that have been made in the aforementioned scales, these range between n = 62 of HACE [14]; HoPE with 77 [15], or n = 134 in the Housing Enabler Screening Tool [13], while in the present HESA II tool, we followed the authors who recommended 20 cases for each factor [62], and we incorporated Cronbach’s alpha and McDonald’s omega reliability indices to obtain a definitive scale that meets the most rigorous standards for the construction process.

Among the limitations of this study, it is noteworthy that the sample is not entirely representative and consists of a small number of participants. Additionally, sampling was only conducted in one autonomous community. Regarding future lines of research, they should focus on using the HESA II tool in different Spanish population groups. Also, they should create an application for use with hardware to enable clinicians to calculate the instrument easily and quickly.

## 5. Conclusions

In Spain, there were no assessment tools for home accessibility adapted to the Spanish context and culture. The HESA II serves to assess home accessibility based on the individual being evaluated rather than on a diagnosis.

It is crucial that a home assessment tool be of high quality, facilitating an accurate assessment involving both healthcare and housing professionals while also engaging and empowering consumers and their caregivers, who may face various functional limitations.

To address these deficiencies, it is essential to integrate the perspective of the International Classification of Functioning, Disability, and Health (ICF), which recognizes that disability is not only a matter of physical barriers but also of personal and environmental factors that affect full participation in society. Therefore, the evaluation of the home environment must consider not only architectural barriers but also aspects such as functional abilities, specific needs, and preferences of each individual. The inclusion of professionals such as occupational therapists in the planning and implementation of accessibility measures can ensure a more complete and person-centered adaptation, thus promoting the independence and quality of life of people with disabilities.

## Figures and Tables

**Table 1:** Descriptive statistics for the study sample.

Variable	Freq.	%
Sex		
Male	51	32.7
Female	105	67.3
Age		
0–13	1	0.64
14–17	6	3.84
18–24	26	16.66
25–34	3	1.92
35–44	5	3.20
45–54	24	15.38
55–64	14	8.97
65–84	49	31.41
>84	24	15.38
Marital status		
Single	43	27.74
Married	64	41.29
Separated/Divorced	3	1.93
Widowed	42	27.09
Income level		
EUR 0–500	5	3.22
EUR 500–1000	17	10.96
EUR 1000–2000	70	45.16
EUR 2000–3000	32	20.64
>EUR 3000	19	12.25
Missing	12	7.74
Area of residence		
Rural	32	20.64
Urban	89	57.41
Centre	23	14.83
Periphery	9	5.80
Missing	3	1.93
Type of residence		
Apartment building	109	70.32
Single-family house	41	26.45
Missing	6	3.87

## Data Availability

Analysis data can be requested from the authors by email.
